# Differences in the Active Endometrial Microbiota across Body Weight and Cancer in Humans and Mice

**DOI:** 10.3390/cancers14092141

**Published:** 2022-04-25

**Authors:** Nadeem O. Kaakoush, Ellen M. Olzomer, Melidya Kosasih, Amy R. Martin, Farokh Fargah, Neil Lambie, Daniella Susic, Kyle L. Hoehn, Rhonda Farrell, Frances L. Byrne

**Affiliations:** 1 School of Medical Sciences, Faculty of Medicine, University of New South Wales, Sydney, NSW 2052, Australia; n.kaakoush@unsw.edu.au; 2 School of Biotechnology and Biomolecular Sciences, Faculty of Science, University of New South Wales, Sydney, NSW 2052, Australia; e.olzomer@unsw.edu.au (E.M.O.); k.hoehn@unsw.edu.au (K.L.H.); 3 School of Women’s and Children’s Health, Faculty of Medicine, University of New South Wales, Sydney, NSW 2052, Australia; melidya.kosasih@health.wa.gov.au (M.K.); amyrose.martin@health.nsw.gov.au (A.R.M.); d.susic@unsw.edu.au (D.S.); 4 Department of Obstetrics and Gynecology, Royal North Shore Hospital, St Leonards, Sydney, NSW 2065, Australia; 5 Laverty Pathology-North Ryde, Macquarie Park, Sydney, NSW 2113, Australia; farokh.fargah@laverty.com.au; 6 Department of Anatomical Pathology, Canterbury Health Laboratories, Christchurch 8011, New Zealand; neil.lambie@cdhb.health.nz; 7 Chris O’Brien Lifehouse, Camperdown, NSW 2050, Australia; rhonda.farrell@lh.org.au; 8 Prince of Wales Private Hospital, Randwick, NSW 2031, Australia

**Keywords:** uterine cancer, microbiota, lactobacillus, obesity

## Abstract

**Simple Summary:**

Of all cancers, endometrial cancer has the greatest association with obesity. Obesity causes dysbiosis of intestinal microbiota, but little is known about whether obesity is associated with dysbiosis of the female genital tract. Therefore, the aim of this study was to determine whether obesity and cancer were associated with altered microbiota profiles in the endometrium. 16S rRNA transcript amplicon sequencing (which captures actively replicating bacteria) of endometrial tissues showed that obesity and cancer were associated with the prevalence of microbial community types in the human endometrium. However, obesity was not associated with microbial community types in the mouse endometrium. The presence of endometrial cancer (but not obesity) was associated with decreased abundance of the *Lactobacillus* genus in the human endometrium. In mice, an enrichment of *Lactobacillus* was associated with lower prevalence of disease (normal uterine histology). These results suggest that obesity and cancer may influence microbiota community types in the endometrium (at least in humans) and *Lactobacillus* may be protective in the endometrium. This study therefore supports further research into the role of microbiota in endometrial cancer development.

**Abstract:**

Obesity is a risk factor for endometrial cancer. The aim of this study was to determine whether actively replicating microbiota in the endometrium differ between obese vs. lean and cancer vs. benign states. We performed 16S rRNA amplicon sequencing on endometrial tissues from lean and obese women with and without endometrial cancer, and lean and obese mice. Results displayed human endometrial microbiota clustered into three community types (R = 0.363, *p* = 0.001). *Lactobacillus* was dominant in community type 1 (C1) while community type 2 (C2) had high levels of *Proteobacteria* and more cancer samples when compared to C1 (*p* = 0.007) and C3 (*p* = 0.0002). A significant increase in the prevalence of the C2 community type was observed across body mass index and cancer (χ^2^ = 14.24, *p* = 0.0002). The relative abundance of *Lactobacillus* was lower in cancer samples (*p* = 0.0043), and an OTU with 100% similarity to *Lactobacillus iners* was enriched in control samples (*p* = 0.0029). Mouse endometrial microbiota also clustered into three community types (R = 0.419, *p* = 0.001) which were not influenced by obesity. In conclusion, obesity and cancer are associated with community type prevalence in the human endometrium, and *Lactobacillus* abundance is associated with normal uterine histologies in humans and mice.

## 1. Introduction

Endometrial cancers arise from the epithelial cells lining the uterus. In 2020, endometrial cancer affected more than 417,000 women and was responsible for more than 97,000 deaths worldwide [1]. Advancing age, late-onset menopause, early menarche, chronic anovulation, unopposed estrogen therapy, tamoxifen treatment, hereditary factors such as Lynch syndrome, and nulliparity are risk factors for this malignancy [2,3,4,5]. Additionally, and what is perhaps of greatest concern given the increasing prevalence of obesity worldwide, is the relationship between obesity and endometrial cancer. In fact, of all malignancies, endometrial cancer has the strongest association with obesity [6]. Calle et al. also reported a 6.25-fold increased risk of uterine (endometrial) cancer-related death for morbidly obese women compared to those with a normal body mass index (BMI) [7]. Mendelian randomization studies also support obesity as a causal factor for endometrial cancer [8,9]. Therefore, identifying specific obesity-related factors that promote endometrial cancer development may provide an opportunity for intervention to reduce risk.

The potential mechanisms linking obesity to endometrial cancer development have been discussed in detail previously [10,11,12,13]. Interestingly, obesity-induced imbalances in estrogen and inflammation can influence microbiota in the gut and vagina [14], and dysbiosis in these environments can promote cancer [14,15]. Recent studies have been interested in determining whether obesity alters microbiota in the uterus/endometrium. One study reported that microbial diversity was significantly increased in endometrial tumors of obese vs. non-obese white women [16]. Another reported that obese women had increased microbial diversity in the lower reproductive tract (cervix and vagina), but BMI was not a significant source of microbial variation within the uterus [17]. Based on these findings, further studies in this field are warranted to determine whether there is a connection between obesity and microbiota of the endometrium. 

The primary aim of this study was to determine whether microbiota profiles differed within the endometrium of lean vs. obese women with and without endometrial cancer. We also examined endometrial microbiota of lean and obese mice. To do this, we performed 16S rRNA transcript amplicon sequencing, which captures actively replicating bacteria of endometrial tissues from women and mice. 

## 2. Results 

### 2.1. Patient Characteristics

We recruited 70 postmenopausal women undergoing hysterectomy for endometrial cancer (endometrioid adenocarcinoma) or benign pathology, either lean or obese. There were four groups: (1) lean women with endometrial cancer (lean cancer, *n* = 17), (2) obese women with endometrial cancer (obese cancer, *n* = 23), (3) lean women with benign disease (lean controls, *n* = 18), and (4) obese women with benign disease (obese controls, *n* = 12). (Table 1). Patient demographics, clinicopathological data, and blood markers are displayed in Table 1.

As expected, the average BMIs for the obese groups (38.20 kg/m^2^ in obese cancer and 31.95 kg/m^2^ in obese controls) were significantly higher (*p* < 0.05) than the lean groups (23.74 kg/m^2^ for lean cancer and 23.65 kg/m^2^ for lean controls) (Table 1). The average ages ranged from 59–69 years across the groups, with the lean cancer group being significantly older than the lean control group on average (69 years vs. 60.5 years, *p* = 0.004). Most endometrial cancers were stage 1A and grade 1, and there were no significant differences in parity, gravidity, age at menarche, age at menopause, or other factors between the groups. Obese women had higher levels of blood glucose, insulin, HbA1c (hemoglobin A1c) International Federation of Clinical Chemistry (IFCC) values, HbA1c National Glycohemoglobin Standardization Program (NGSP) (%) values, C-reactive protein (CRP), and estradiol (Table 1).

### 2.2. Human Endometrial Microbiota Is Altered in Cancer

To examine the endometrial microbiota, 16S rRNA transcript amplicon sequencing (i.e., cDNA sequencing) was performed as described in Methods and previously [18]. This method captures actively replicating bacteria and has advantages compared to sequencing DNA because it reduces potential bias from dead microbes, relic DNA, and contaminants [19,20], while still identifying similar taxa [21]. We also included six negative controls to reduce potential signals from contaminants. These included buffers from the Allprep kit (RPE, RW1 and lysis buffer), two master mix solutions from the cDNA reactions (without reverse transcriptase, one each for the human and mouse cohorts), and water that was used in all reactions. Results demonstrated that the number of OTUs was significantly lower in the negative control samples when compared to human and mouse endometrial samples (Figure S1A). Furthermore, principal coordinate (PCO) and non-metric multidimensional scaling plot (nMDS) analyses of beta diversity (microbial diversity across groups) illustrated significant differences (*p* = 0.001) between controls vs. human endometrial samples (Figure S1B,C) and controls vs. mouse endometrial samples (Figure S1D,E), indicating robust profiling of the active endometrial microbiota and minimal influence of environmental contamination. 

The bacterial component of the endometrial microbiota was then examined in patient samples to identify microbial signatures that may be associated with obesity and carcinogenesis. For women with cancer, we only included tumor samples to avoid duplication of that patient. Of the 70 women recruited to the study, 65 patients were included in these analyses from each of the four groups: (1) lean cancer (*n* = 14), (2) obese cancer (*n* = 21), (3) lean controls (*n* = 18), and (4) obese controls (*n* = 12). The remaining five patients had insufficient samples for these analyses. Notably, the majority (89%) of patients were Caucasian, with *n* = 12 Caucasian and *n* = 2 Asian in the lean cancer group; *n* = 19 Caucasian, *n* = 1 Asian, and *n* = 1 unknown in the obese cancer group; *n* = 16 Caucasian, *n* = 1 Asian, and *n* = 1 unknown in the lean control group; and *n* = 11 Caucasian, and *n* = 1 Asian in the obese control group. 

**Figure 1:**
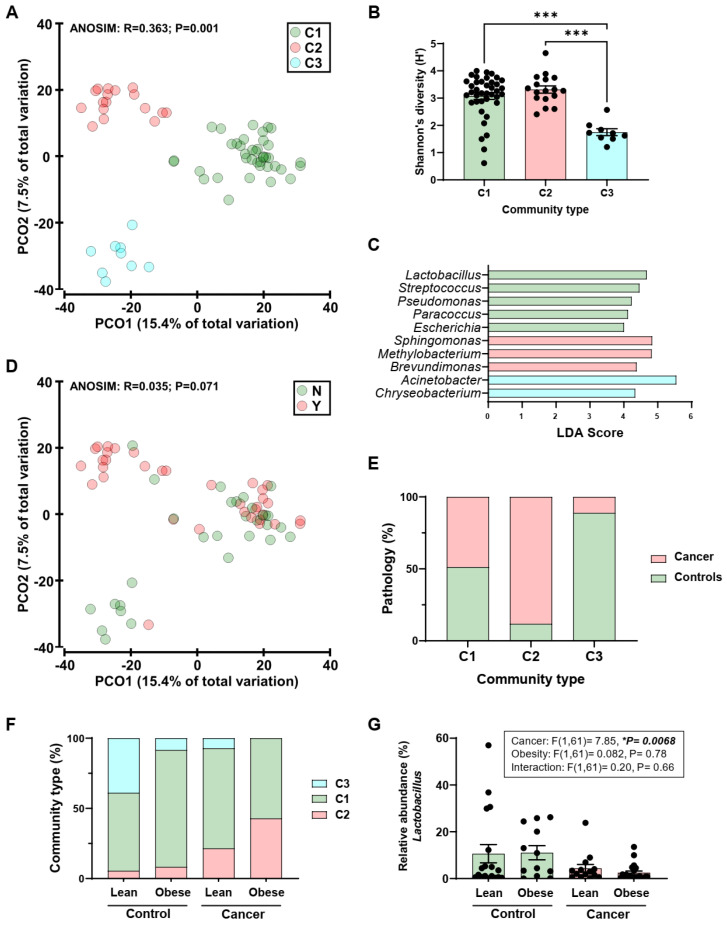
Endometrial microbiota of patients with endometrial cancer and controls. (**A**): Principal coordinate (PCO) analysis of bacterial beta diversity across community types (C1-3). The ordinations were generated from Bray-Curtis resemblance matrices on square-root transformed bacterial relative abundances. Analysis of similarities (ANOSIM) was employed to test for significant differences in beta diversity. (**B**): Shannon’s diversity across community types. Significance was analyzed by Kruskal-Wallis with Dunn’s multiple comparisons test. *** *p* < 0.001. (**C**): Genera found to be significantly differentially abundant across community types. Taxa were identified using Linear discriminant analysis Effect Size (LEfSe). Genera with a Linear discriminant analysis (LDA) score > 4 are displayed. (**D**): Principal coordinate analysis of bacterial beta diversity between patients (Y, tumor sample) and controls (N). (**E**): Prevalence (%) of patients with endometrial cancer across community types. (**F**): Prevalence (%) of community types in patients and controls stratified according to body mass index (BMI). (**G**): Relative abundance (%) of *Lactobacillus* in patients and controls stratified according to BMI. Significance was tested using two-way ANOVA, * *p* < 0.01.

Results demonstrated that the endometrial microbiota clustered into three community types (Figure 1A) that were significantly different in composition (R = 0.363, *p* = 0.001; ANOSIM). Canonical analysis of principal coordinates had a 100% concordance (65/65) with the classification of samples into community types. In further support of the classification, the community types had significant differences in alpha diversity (microbial diversity within each sample) measures (Figure 1B and Figure S2) and significantly differentially abundant taxa (Figure 1C). Community type 1 (C1) had a dominance of lactic acid-producing bacteria such as *Lactobacillus* and *Streptococcus*, while community type 2 (C2) had high levels of *Proteobacteria* (alpha and beta) (Figure 1C). Community type 3 (C3) had *Acinetobacter* and *Chryseobacterium* as dominant bacteria (Figure 1C).

Examination of beta diversity in endometrial samples from women with cancer and women without cancer (controls) demonstrated that there were no significant differences (R = 0.035, *p* = 0.071; ANOSIM) (Figure 1D). However, the ordination plot was suggestive of differential prevalence of cancer and control samples across community types (Figure 1D, Y = tumor samples, N = controls), and this was confirmed upon further inspection (χ^2^ = 15.11, *p* = 0.0001; Chi-square for trend; Figure 1E). Specifically, a significantly higher prevalence of cancer samples was identified in C2 when compared to C1 (*p* = 0.007, Fisher’s exact) and C3 (*p* = 0.0002, Fisher’s exact). To identify taxa enriched or depleted in cancer samples, the relative abundance of taxa in cancer samples were compared to those in controls while correcting for community type. One taxon, *Lactobacillus* OTU23, with 100% similarity to *Lactobacillus iners*, was identified to be significantly enriched in control samples [Linear discriminant analysis (LDA) score = 4.5, *p* = 0.0029].

### 2.3. Obesity Does Not Determine Lactobacillus Abundance in the Uterus

To establish if obesity was associated with changes in microbiota, patients were classified according to their body mass index (BMI), and the prevalence of community types within each group was assessed. A significant increasing trend of the C2 community type was observed across BMI and cancer (χ^2^ = 14.24, *p* = 0.0002; Chi-square for trend; Figure 1F), suggesting obesity may play a confounding role in the context of community type prevalence. However, the relative abundance of the *Lactobacillus* genus was influenced by the presence of cancer and not classification according to obesity (Cancer: F(1,61) = 7.85, *p* = 0.0068, Obesity: F(1,61) = 0.082, *p* = 0.78, Interaction: F(1,61) = 0.20, *p* = 0.66, Figure 1G).

### 2.4. Endometrial Tumors and Adjacent Normal Endometrium Share Similar Microbiota

**Figure 2:**
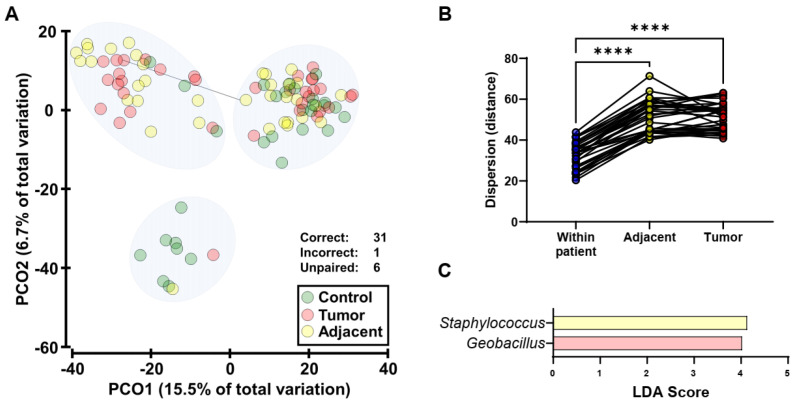
Endometrial microbiota in tumor samples and healthy adjacent tissue. (**A**): Principal coordinate (PCO) analysis of bacterial beta diversity across sample types (control, green; healthy adjacent tissue, yellow; tumor tissue, red). The ordinations were generated from Bray-Curtis resemblance matrices on square-root transformed bacterial relative abundances. All paired tumor-adjacent samples except one (indicated by connecting line) classified as the same community type. (**B**): Comparisons of dispersions in microbiota composition within patients (tumor-adjacent) against inter-patient (adjacent and tumor). Distances were calculated using the distance-based test for homogeneity of multivariate dispersions (PERMDISP). Significance was tested by ANOVA using Tukey’s multiple comparisons test. **** *p*< 0.0001. (**C**): Genera that were significantly differentially abundant between tumor samples and adjacent normal tissue. Taxa were identified using Linear discriminant analysis Effect Size (LEfSe). Genera that had a Linear discriminant analysis (LDA) score > 4 are displayed. No taxa were identified when correction for community type was applied.

Differences in the microbiota between tumor samples and healthy adjacent tissue from patients with endometrial cancer were examined. The majority, 31/32, (96.9%) of paired tumor and adjacent samples classified as the same community type (Figure 2A), indicating a lack of substantial shifts between the two sample types within patients. In support of this, the distances between samples within patients were calculated and compared across patients in both tumor and adjacent tissue, and significantly higher distances were calculated inter-patient compared to intra-patient (Figure 2B). Furthermore, minimal differences in differential taxa were identified between tumor and adjacent tissue (Figure 2C), and no taxa were identified when correction for community type was applied. 

### 2.5. Western Diet-Induced Mouse Model of Obesity 

Next, we sought to investigate whether obesity alters uterine microbiota in mice. Liver kinase B1 (*LKB1*) is a tumor suppressor in endometrial cancer [22,23] with expression lost in 20–50% of sporadic endometrial cancer cases [24,25]. Researchers previously reported that Cre-induced knockout of both LKB1 (mouse gene *Stk11*) alleles in the endometrium of mice (*Sprr2f-Cre; Lkb1^L/L^*) promotes the development of highly invasive adenocarcinomas in 100% of mice [23]. In contrast, mice that lacked one copy of LKB1 in the endometrium *(Sprr2f-Cre; Lkb1^L^^/+^*) were weakly susceptible to endometrial cancer, with one out of 12 mice developing a focal endometrial tumor at ~600 days old [23]. We backcrossed the LKB1 floxed and Sprr2f-Cre mice onto a C57BL/6J background to minimize strain-specific effects of the mixed FVB and 129 backgrounds of the initial founder mice. The C57BL/6 strain was chosen because it is more susceptible to diet-induced obesity [26,27]. Importantly, the cancer phenotype observed in *Sprr2f-Cre; Lkb1^L/L^* female mice on the FVB;129 mixed background was maintained in C57BL/6J female mice (Figure S3A–C).

**Figure 3:**
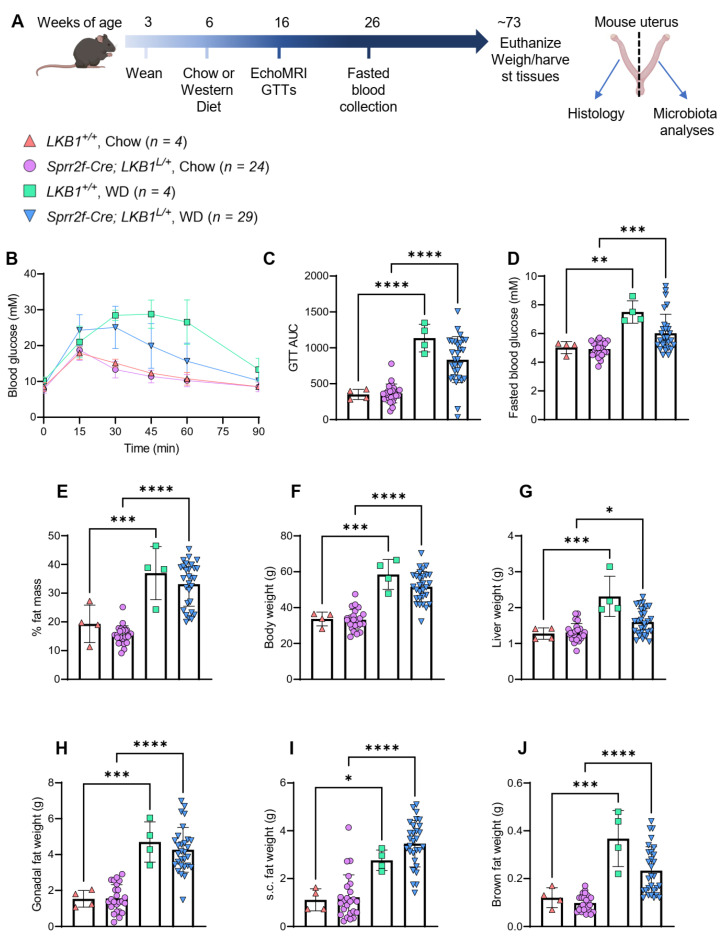
Long-term Western diet-induced model of obesity in female mice. (**A**)**:** Schematic overview of mouse study, where female mice lacking one copy of liver kinase B1 (*LKB1*) in the endometrium (*Sprr2f-Cre: LKB1^L^*^/+^) or wild-type littermates (*LKB1^+^*^/+^) were weaned at 3 weeks of age, fed chow diet until 6 weeks of age, and then continued either on chow or Western diet (WD) at 6 weeks of age. Fat mass was measured by EchoMRI and glucose tolerance tests (GTTs) were performed at 16 weeks of age. Blood samples were collected following a 17-h fast at 26 weeks of age. Mice were euthanized at ~73 weeks of age (~510 days old). Mice uteri were cut in half for histological and microbiota analysis. (**B**): Glucose tolerance test (GTT), whereby mice were injected with 2 g/kg glucose (intraperitoneal, *i.p.*) and their blood glucose levels (millimolar, mM) were measured over time. (**C**): Area under the curve values from the GTT for each of the 4 groups. (**D**): Blood glucose levels (mM) were measured in mice following a 17 h fast. (**E**): Percentage (%) fat mass of mice was measured using echo magnetic resonance imaging (MRI). (**F**): Final mouse body weights at study completion. (**G)**: Liver weights of mice at study completion. (**H**): Gonadal fat weights (both pads) of mice at study completion. (**I**): Subcutaneous fat weights (both pads) of mice at study completion. (**J**): Brown fat weights of mice at study completion. Mouse numbers for each group are displayed next to the symbols, with *n* = 4 for *LKB1^+^*^/+^ on chow and Western diets, *n* = 24 for *Sprr2f-Cre: LKB1^L^*^/+^ mice on chow diet, and *n* = 29 for *Sprr2f-Cre: LKB1^L^*^/+^ mice on Western diet (WD). Mouse data was analyzed by 2-way ANOVA with Tukey’s test for multiple comparisons (alpha = 0.05). Data bars indicate the mean and error bars indicate standard deviation. * *p* < 0.05, ** *p* < 0.01, *** *p* < 0.001, **** *p* < 0.0001.

Mice that lacked one copy of LKB1 in the endometrium (*Sprr2f-Cre; Lkb1^L^^/+^*) were used determine whether obesity promotes endometrial cancer in this model, and to examine the relationship between obesity and uterine microbiota in mice. Female *Sprr2f-Cre; Lkb1^L^^/+^* mice fed Western diets were compared to mice of the same genotype fed chow diets (Figure 3A). We also included a small number (*n* = 4) of wild-type littermates (that had both copies of the LKB1 allele in the endometrium, *Lkb1^+^^/+^*) as additional controls (Figure 3A). Mice were weaned at 3 weeks of age and fed a chow diet until 6 weeks of age. Mice then either continued a chow diet or began a Western diet until they were ~73 weeks old (average age of 510 days old) (Figure 3A). This time point was chosen because we wanted the mice to be at early reproductive senescence (as per our human cohort). We estimated that 510-day-old mice would be ~58 in human years [28], which is ~6 years after the average age of menopause in humans.

Metabolic analyses confirmed that all mice (both genotypes) fed a Western diet had poorer glucose clearance after a glucose bolus challenge (Figure 3B,C), higher fasting blood glucose (Figure 3D), higher body fat mass (Figure 3E), and higher body weight (Figure 3F), liver weight (Figure 3G), gonadal fat weight (Figure 3H), subcutaneous fat weight (Figure 3I), and brown fat weight (Figure 3J) compared to mice fed the chow diet. Therefore, Western diet feeding resulted in the expected effects of obesity and obesity-related metabolic impairment. However, histological analyses found no change in uterine pathology, with similar percentages of mice having normal histology, hyperplasia, and atypical hyperplasia between the chow vs. Western diet groups for both genotypes (Chi-square = 0.95, *p* = 0.62, Table 2). Approximately half of all mice (31/61) had atypical hyperplasia but only two mice (one each from the chow and Western diet groups) had cancer (grade 1/stage 1A endometrial adenocarcinoma). Therefore, obesity did not promote endometrial cancer development or hyperplasia/atypical hyperplasia in C57BL/6J mice, regardless of genotype.

### 2.6. Mouse Endometrial Microbiota 

**Figure 4:**
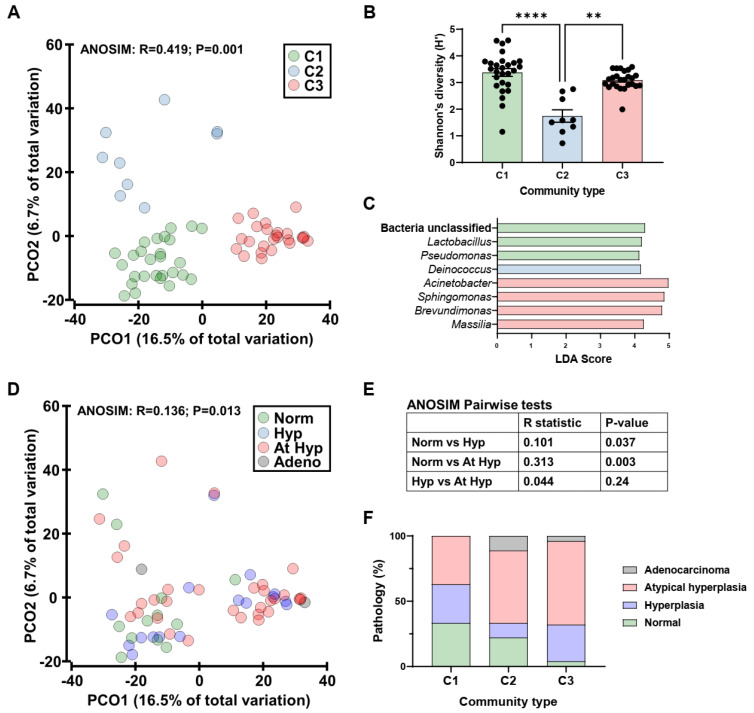
Endometrial microbiota of mice on chow or a western diet. (**A**): Principal coordinate (PCO) analysis of bacterial beta diversity across community types (C1-3). The ordinations were generated from Bray-Curtis resemblance matrices on square-root transformed bacterial relative abundances. Analysis of similarities (ANOSIM) was employed to test for significant differences in beta diversity. (**B**): Shannon’s diversity across community types. Significance was tested using Kruskal-Wallis with a Dunn multiple comparisons test. ** *p* < 0.01, **** *p* < 0.0001. (**C**): Genera found to be significantly differentially abundant across the community types. Taxa were identified using Linear discriminant analysis Effect Size (LEfSe) and only genera that had a linear discriminant analysis (LDA) score > 4 are displayed. (**D**): Principal coordinate analysis of bacterial beta diversity across pathological outcomes. Norm: normal, Hyp: hyperplasia, At Hyp: atypical hyperplasia, adeno: adenocarcinoma. (**E**): Results of pairwise ANOSIM tests. (**F**): Prevalence of pathological outcomes across community types.

The actively replicating bacterial component of the endometrial microbiota was then profiled in lean and obese mice (of both genotypes) using 16S rRNA transcript amplicon sequencing. Uterine microbiota of mice clustered into three community types (C1-3, Figure 4A) that were significantly different in composition (R = 0.419, *p* = 0.001; ANOSIM). The community types were different in their alpha diversity measures, driven by species evenness (Figure 4B and Figure S4A,B). Analysis of taxa that were differentially abundant across the community types identified C1 to be enriched with *Lactobacillus* and *Pseudomonas*, C2 with *Deinococcus,* and C3 with *Acinetobacter*, *Sphingomonas*, *Brevundimonas,* and *Massilia* (Figure 4C). Canonical analysis of principal coordinates had a high concordance with the classification of samples into community types (96.7%, 59/61 samples).

To assess if these community types were associated with pathological outcomes, microbiota composition was assessed against the four outcomes (normal, hyperplasia, atypical hyperplasia, and adenocarcinoma) (Figure 4D), and a significant relationship was observed (R = 0.136, *p* = 0.013; ANOSIM). Pairwise analysis revealed that this relationship was driven by differences between normal and hyperplastic tissues in addition to normal and atypical hyperplastic tissues (Figure 4E). The prevalence of each pathological outcome within each community type was then assessed, which suggested an increasing prevalence of disease from C1 to C3 (Figure 4F). The prevalence of normal vs. disease (hyperplasia, atypical hyperplasia, and adenocarcinoma) was compared across community types which displayed a trend towards a difference (χ^2^ = 3.65, *p* = 0.056; Chi-square for trend), which was due to differences between C1 and C3 (*p* = 0.012, Fisher’s Exact). The community types in the mouse uteri were not due to differences in diet or body weight at endpoint (Figure S4C,D).

## 3. Discussion

Obesity is a major risk factor for endometrial cancer, particularly endometrioid adenocarcinomas that account for most endometrial cancer cases [29]. Although this cancer type typically favors a good prognosis, there remains an increased risk of cancer-related death, particularly among morbidly obese women [7], and risks associated with surgery [30]. As such, identifying obesity-related factors that may promote cancer development in obese women is an important step forward in developing strategies to reduce cancer risk. 

This study found obesity and cancer were associated with the prevalence of microbial community types in the human endometrium. Previous studies have also indicated that obesity may be associated with microbiota changes in the endometrium. One study (published conference abstract) noted a potential trend (*p* = 0.07) towards a difference in uterine microbial composition between obese and non-obese (BMI < 30 kg/m^2^) Caucasian women with endometrial cancer [31]. The same group recently reported that microbial diversity was significantly increased in endometrial tumors of obese vs. non-obese white women [16]. Furthermore, the abundance of Firmicutes was significantly higher, while the abundance of OD1 was significantly lower, in endometrial tumors of non-obese vs. obese white women [16]. Another study by Walsh et al. found that obese women had increased microbial diversity in the lower reproductive tract (cervix and vagina), but BMI was not a significant source of microbial variation within the uterus [17]. Therefore, obesity could be playing some role in shaping the microbiota of the endometrium. However, in our mouse model, we found that diet-induced obesity was not associated with microbial community types within the endometrium. Therefore, further studies in humans are required to determine whether obesity-related microbiota changes influence cancer development in the endometrium.

*Lactobacillus* are thought to play a protective role in the female reproductive tract. In the lower reproductive tract, a dominance of *Lactobacillus* species, including *L. crispatus*, *L. gasseri*, and *L. jensenii,* may protect against pathogens because they produce lactic acid (via metabolism of glycogen byproducts) and bacteriocins, and prevent pathogen adhesion to the vaginal epithelium via competitive exclusion [14]. Dysbiosis of vaginal microbiota, whereby *Lactobacillus* are depleted, and diverse anaerobes dominate, is associated with increased risk of preterm birth, sexually transmitted infections, miscarriages, and gynecological cancers, including cervical cancer [14,32]. *Lactobacillus* presence in the uterus may also be a marker of reproductive health, as reviewed [33,34]. In our study we found that women with cancer had significantly lower levels of *Lactobacillus* in the endometrium compared with women without cancer (controls). Therefore, higher amounts of *Lactobacillus* in the uterus may be associated with lower cancer risk. However, it is important to note that we also identified other bacteria enriched in the C1 community type alongside *Lactobacillus*, including *Streptococcus*, *Pseudomonas*, *Paracoccus*, and *Escherichia*. The other two community types were enriched with *Sphingomonas*, *Methylobacterium*, and *Brevundimonas* (C2); and *Acinetobacter* and *Chryseobacterium* (C3). Interestingly, the C3 community type enriched with *Acinetobacter* had the most control samples, i.e., the lowest cancer samples, suggesting that *Acinetobacter* may be associated with a normal (or at least benign) endometrium. In support of this, a study by Winters et al. reported that the endometria of women with a median age of 45 (who underwent hysterectomy for fibroids) were dominated by *Acinetobacter* and not *Lactobacillus*, with a single *Acinetobacter* OTU having over 60% relative abundance in the endometrium [35]. Another study by Chen et al. reported that the uteri of women of reproductive age were dominated by *Lactobacillus* (30.6%), *Pseudomonas* (9.09%), and *Acinetobacter* (9.07%) [36]. Therefore, our study results complement the findings of others in this field. However, more research is required to understand the roles of *Lactobacillus* and *Acinetobacter* in uterine health, and whether their abundance is impacted by factors like menopause and cancer. 

Our human data revealed that one taxon, similar to *Lactobacillus iners*, was significantly enriched in the endometria of women without cancer vs. women with cancer. Our results support a study by Walsh et al., which reported that two taxa similar to *L. iners* were depleted in endometrial cancer tissues compared to benign tissues [17]. However, Winters et al. reported that *L. iners* was rarely detected in endometria of women with an average age of 45 years [35]. These studies further highlight the controversies surrounding the role of *L. iners* in the female reproductive tract [37,38]. 

Of the four most common vaginal *Lactobacillus* species, only *L. iners* is unable to synthesize D-lactic acid (due to the absence of the gene-encoding D-lactate dehydrogenase) and instead produces L-lactic acid [32]. L-lactic acid is thought to be less protective against vaginal dysbiosis and infections than D-lactic acid [39]. Furthermore, *L. iners* is unable to produce hydrogen peroxide which, through hydroxyl free radical production, inhibits catalase-negative anaerobic bacteria [32]. A dominance of *L. iners* in the cervix was associated with obesity in premenopausal Korean women [40]. However, this study defined obesity as a BMI of ≥25 kg/m^2^, and the association between *L. iners* and obesity was not observed in postmenopausal women. Nevertheless, *L. iners* is a normal inhabitant of the female reproductive tract [41] and is thought to be a transitional species that colonizes the vagina following disruption [37]. Other studies suggest that *L. iners* is not pathogenic, and in fact may be important for host defense and restoring the vaginal microbiota via production of anti-inflammatory and antimicrobial molecules [37]. It is therefore unclear whether *L. iners* plays differing roles in the epithelium of the lower vs. upper female reproductive tract. 

Many human pathogens are from the *Proteobacteria* phylum, including *Helicobacter pylori*, which is a risk factor for non-cardia gastric cancer. *Proteobacteria* are Gram-negative bacteria with lipopolysaccharide (LPS) in the outer membrane, and LPS is an endotoxin that induces a strong inflammatory response [42]. In our study, *Proteobacteria* including *Sphingomonas*, *Brevundimonas,* and *Methylobacterium* (in humans) and *Sphingomonas*, *Brevundimonas, Acinetobacter*, and *Massilia* (in mice) were associated with uterine pathologies. Other studies support a role for *Proteobacteria* in reproductive failure and endometriosis, in addition to endometrial and ovarian cancers [33,43,44,45]. *Proteobacteria* was the most abundant phyla, with an enrichment of *Sphingomonas*, *Methylobacterium,* and *Acinetobacter* genera in ovarian cancer tissues compared to normal distal fallopian tubes [46]. Whether these bacteria contribute to an inflammatory microenvironment within the uterus and cancer development remains to be determined. 

Analysis of the microbiota in mouse endometrial samples revealed three distinct community types. However, these were not associated with Western diet or mouse body weights. Therefore, obesity was not associated with endometrial microbial community types in mice. The differences we observed between the mouse and human data could be attributed to several factors. In particular, the conditions driving obesity in mice were better controlled, e.g., mice of the same strain were fed the same diets (chow or Western diet) for a similar period, whereas in humans, the onset of obesity could occur at different times and be impacted by different diets and a sedentary lifestyle. However, like in humans, an enrichment of *Lactobacillus* genera was associated with lower prevalence of disease (normal uterine histology), while *Proteobacteria* genera were associated with uterine pathologies in mice. Interestingly, *Sphingomonas* and *Brevundimonas* were both enriched in human (C2) and mouse (C3) community types and were associated with the presence of cancer and uterine pathologies (hyperplasia/atypical hyperplasia). Therefore, these bacteria may be associated with uterine pathologies across mammalian species. However, it is important to note that the OTUs within those genera (driving the community types in humans and mice) had sequence similarities to distinct species i.e., it is likely these were different species. 

Evaluation of uterine microbiota could have been at risk of contamination due to sampling technique, e.g., collection via the cervicovaginal canal, or external contaminants during processing [35,47]. We reduced the potential risk of contamination by collecting endometrial tissue immediately following hysterectomy (under sterile conditions), including controls in our sequencing to exclude potential contamination from kit reagents (also referred to as the “kitome” [35]), and profiling viable bacteria by sequencing cDNA from DNase-treated RNA. This method, compared with sequencing DNA, captures actively replicating bacteria and reduces potential bias from dead microbes, relic DNA, and contaminants, and is well suited to samples with a lower abundance of microbes [19,20]. Indeed, several studies have indicated there are approximately 10,000-fold fewer bacteria in the upper female reproductive tract compared to the lower reproductive tract [14,35]. To our knowledge, ours is the first to study to investigate the “active” microbiota of the endometrium. Furthermore, our study complements that of others in this field to gain a broader understanding of microbiota in the endometrium. 

A previous study reported that microbial diversity was the same in the mid-endometrium vs. the whole endometrium within patients [35]. However, no study has yet compared the microbiota of endometrial tumor tissue vs. adjacent non-tumor tissue. Our study found microbiota profiles were similar between matched tumor vs. non-tumor endometrial tissue within patients. These results suggest that the presence of the tumor itself does not impact the local microbiota, and that changes to endometrial microbiota community types (that are observed between patients with and without cancer) may occur prior to tumorigenesis. However, further studies are required to determine whether dysbiosis of the uterus/endometrium causes cancer development in this environment. 

A limitation of our study was that our controls were from patients with benign conditions and thus, samples were not necessarily from a normal endometrium. Furthermore, while we report that no patients were taking antibiotics in the 3 weeks prior or at the time of surgery, we did not have information on antibiotic intake prior to that. Nevertheless, other studies in this field have excluded patients who had taken antibiotics within 2 weeks of surgery [43] or 10 days prior to surgery [35]. 

## 4. Methods

### 4.1. Patient Cohort and Ethics

Women were recruited to our clinical study at the Royal Hospital for Women/Prince of Wales Private Hospital (Randwick, Sydney, Australia) from 4 April 2016 until 20 June 2018, with the inclusion criteria of: 18 years or older, postmenopausal (ceased having regular periods at least 12 months prior), body mass index (BMI) of either <27 kg/m^2^ (lean) or >30 kg/m^2^ (obese), and planned hysterectomy for benign disease or endometrial cancer (diagnosis of endometrioid adenocarcinoma on currettings, any grade). A cutoff of less than 27 kg/m^2^ was used for the lean groups to overcome difficulties in recruiting sufficient patients with BMI less than 25 kg/m^2^. However, the average BMI of the lean groups was below 25 kg/m^2^ (Table 1). Exclusion criteria were premenopausal women, BMI within the range of 27-30 kg/m^2^, recipient of an investigational new drug within prior 6 days (with an unknown action), and history of psychological illness or condition that may interfere with patient’s ability to understand the requirements of the study. No women in this study were taking antibiotics at the time of surgery or in the 3 weeks prior to surgery. The final numbers of patients (total, *n* = 70) in each group were as follows: (1) lean cancer (*n* = 17), (2) obese cancer (*n* = 23), (3) lean controls (*n* = 18), and (4) obese controls (*n* = 12). Consent was received from all patients prior to sample collection, and all processing and experiments were approved by the Human Research Ethics Committee (HREC) of the South Eastern Sydney Local Health District (HREC 15/339). Clinical data was recorded in a de-identified database and matching samples were stored at the Lowy Biobank UNSW, Sydney.

### 4.2. Collection of Human Blood and Endometrial Tissues

Patient blood samples were obtained on the day of surgery following a period of fasting from food for 8 h and clear fluids up to 2 h prior to hysterectomy. Blood samples were collected in BD vacutainers K2E (EDTA) (Cat# 367525) and sent to SEALS Pathology (Prince of Wales Hospital) for clinical chemistry testing. Endometrial tissues were collected immediately following hysterectomy by surgically opening the uterus in a sterile environment, which is thought to reduce the risk of bacterial contamination from the cervix [14]. Benign endometrial tissue was obtained from women without cancer, and benign and adjacent malignant endometrial tissue was collected from women with cancer. Intact uteri were immediately placed on ice and delivered to pathology for processing. Sections of benign or malignant endometrial tissue were collected by a pathologist under sterile conditions and immediately placed in 1 mL of Allprotect tissue reagent (Qiagen) in sterile cryovial tubes, and then stored at -80 °C until processed. 

### 4.3. Mouse Diet Study

All mouse experiments were carried out in accordance with relevant guidelines and regulations approved by the UNSW Animal Care and Ethics Committee (project approval #14/33A). Liver kinase B 1 (LKB1) (gene name in mice *Stk11*), floxed mice (FVB;129S6-*Stk11^tm1Rdp^*/Nci), and Sprr2f-Cre mice (FVB-Tg(Sprr2f-cre)1Dcas/Nci) were obtained from the National Cancer Institute (USA) and maintained in pathogen-free vivaria at the University of New South Wales (Australia). Male mice (*Sprr2f-Cre; Lkb1^L^^/+^*) were backcrossed with pure C57BL/6J female mice (4 generations) to create mice that were >90% C57BL/6. All mice used in this study were littermate females that either lacked one copy of LKB1 in the endometrium (*Sprr2f-Cre; Lkb1^L^^/+^*) or were wild-type (had both copies) of the LKB1 allele (*Lkb1^+^^/+^*). When mice reached 6 weeks of age, they were fed either a healthy chow diet (#17 Premium Rat & Mouse-Irradiated, Gordons Specialty Feeds, NSW, Australia) or Western diet prepared in-house which contained 45% fat and 16% sucrose by kCal (based on Research Diets Inc. rodent diet D12451), as previously described [39,48]. Mice were housed (maximum 5/cage) at the Biological Resources Centre (UNSW) and had access to food and water *ad libitum* in 12-h light/dark cycles. Mice were culled on average at 73 weeks of age (~510 days old) by cervical dislocation following CO_2_ asphyxiation. Tissues were collected, weighed, and fixed for histology (in 10% formalin solution) or snap-frozen in liquid nitrogen. 

### 4.4. Mouse Genotyping

DNA was extracted from mouse tail tips using the HotSHOT method [49]. Genomic DNA was amplified using MyTaq DNA polymerase (Bioline) with 400 nM primers. LKB1 primer sequences were PCRS5 (5′-tctaacaatgcgctcatcgtcatcct-3′), LKB36 (5′-gggcttccacctggtgccagcctgt-3′), and LKB39 (5′-gagatgggtaccaggagttggggct-3′). Cre primer sequences were NA1 (5′-ggtacacacgtcctggaatac-3′) and NA2 (5′-ttcccattctaaacaacaccctgaa-3′). PCR conditions for Cre were as follows: [94 °C (3 min), 35 cycles [94 °C (30 s), 65 °C (30 s), 72 °C (30 s)], 72 °C (3 min)] and for LKB1: [94 °C (3 min), 35 cycles [94 °C (30 s), 60 °C (30 s), 72 °C (60 s)], 72 °C (3 min)]. Samples were run on 1.5% agarose gels in TAE buffer and DNA bands were visualized with ethidium bromide on a Bio-Rad Gel Doc.

### 4.5. Mouse Metabolic Analyses 

Glucose tolerance tests (GTTs) were conducted on all mice at 16 weeks of age, following i.p. injection of 2 g/kg glucose solution (prepared at 333 mg/mL in sterile 0.9% saline solution). Fat mass (% of total body weight) was measured using an EchoMRI when mice were 16 weeks of age. Blood glucose levels were measured in mice at 26 weeks of age following a 17-h fast from food. 

### 4.6. Mouse Uterine Pathology

Mouse uteri were inspected visually for macroscopic tumors before cutting in half (1 uterine horn each side) under sterile conditions. One half of the uterus was collected for pathology (fixed in 10% formalin solution) and the other half snap frozen for analysis of uterine microbiota. Fixed uteri were then sectioned, placed on glass slides, and stained with H&E. Slides were then analyzed by a certified pathologist in a blinded manner (Laverty Pathology, Sydney, Australia). 

### 4.7. 16S rRNA Transcript Amplicon Sequencing and Analysis

Mouse and human endometrial tissues were powdered, and RNA was extracted using an AllPrep DNA/RNA/Protein Mini Kit (Qiagen, Cat # 80004). cDNA was synthesized from 100 ng of DNase-treated RNA using a High-Capacity cDNA reverse transcription kit (Applied Biosystems, Cat # 4368813). The V4 region of the 16S rRNA region was profiled using Illumina MiSeq 2 × 250 bp chemistry as previously described [50]. Raw reads were analyzed using Mothur v1.39.1 (human) and v1.44.0 (mouse) [51] and the MiSeq standard operating procedures with some minor modifications [52]. Negative controls were buffers from the Allprep kit (RPE, RW1 and lysis buffer), two master mix solutions (without reverse transcriptase) from the cDNA reactions, and water that was used in all reactions. Operational taxonomic units (OTUs) that were detected in the negative controls were not considered if identified as a differential feature in any comparison. Read depth for 16S rRNA data from the human and mouse cohorts were 2793 and 6781 clean reads/sample after subsampling. 

### 4.8. Statistical Analyses

Human patient information (Table 1) was analyzed in SPSS and pairwise comparisons were performed between each group for all data points. P values were calculated using a Kruskal-Wallis H Test and were adjusted for multiple comparisons using the Bonferroni correction for multiple comparisons test. For microbiota analyses, α-diversity measures (Margalef’s species richness, Pielou’s species evenness and Shannon’s diversity index), Bray-Curtis dissimilarities, Principal Coordinate Analysis (PCoA), Analysis of Similarities (ANOSIM), and Permutational analysis of multivariate dispersions (PERMDISP) were conducted using Primer-E v6 software. Per taxon analyses were performed using LEfSe [53]. All other tests were performed in GraphPad Prism v9. Normality was tested using the Shapiro–Wilk test, and alpha diversity measures that were not normally distributed were analyzed using the Kruskal-Wallis (KW) test. Mouse data was analyzed in GraphPad Prism v9 using two-way ANOVA with Tukey’s test for multiple comparisons (alpha = 0.05). 

## 5. Conclusions

This study found that obesity and cancer were associated with the prevalence of microbial community types in the human endometrium, but Western diet-induced obesity was not associated with microbial community types in the mouse endometrium. The presence of endometrial cancer (but not obesity) in women was associated with decreased abundance of the *Lactobacillus* genus in the endometrium. Furthermore, *Lactobacillus* OTU23 (100% similarity to *Lactobacillus iners*) was enriched in endometrial samples from women without endometrial cancer. In mice, an enrichment of *Lactobacillus* was associated with lower prevalence of disease (normal uterine histology). These results suggest that obesity and cancer may influence microbiota community types in the endometrium (at least in humans) and *Lactobacillus* may be protective in the endometrium, like in the lower female reproductive tract. This study therefore supports further research into the role of microbiota in endometrial cancer development.

## Figures and Tables

**Table 1:** Baseline characteristics and metabolic parameters of patients.

Characteristics: Median or n (%)	Lean Cancer (*n* = 17)	Obese Cancer (*n* = 23)	Lean Control (*n* = 18)	Obese Control (*n* = 12)	*p*-Value ^m^
Age (years)	69.0 ^a^	68.0	60.5 ^a^	59.5	0.003
BMI (kg/m^2^)	23.74 ^b^	38.20 ^b^	23.65 ^b^	31.95 ^b^	<0.0001
Stage					
I A	10 (58.8%)	15 (65.2%)	N/A	N/A	
I B	4 (23.5%)	3 (13%)			
II A	0 (0.0%)	1 (4.3%)			
III A	0 (0.0%)	1 (4.3%)			
III C	1 (5.9%)	2 (8.7%)			
IV A	1 (5.9%)	0 (0.0%)			
IV B	1 (5.9%)	1 (4.3%)			
Grade			N/A	N/A	
1	13 (76.5%)	14 (60.9%)			
2	3 (17.6%)	8 (34.8%)			
3	1 (5.9%)	1 (4.3%)			
Parity					
0	5 (29.4%)	6 (26.1%)	6 (33.3%)	3 (25.0%)	
1 or 2	9 (52.9%)	6 (26.1%)	8 (44.4%)	6 (50.0%)	
3 or more	3 (17.6%)	11 (47.8%)	4 (22.2%)	3 (25.0%)	
Gravidity					
0	4 (23.5%)	7 (30.4%)	4 (22.2%)	2 (16.7%)	
1 or 2	8 (47.1%)	4 (17.4%)	11 (61.1%)	6 (50.0%)	
3 or 4	2 (11.8%)	10 (43.5%)	3 (16.7%)	3 (25.0%)	
5 or more	3 (17.6%)	2 (8.7%)	0 (0.0%)	1 (8.3%)	
Age at Menarche (years)	13.5 (*n* = 16)	13.0	13.0 (*n* = 17)	13.0	0.567
Age at Menopause (years)	51.0	52.0	52.0 (*n* = 15)	50.5	0.681
Any systemic HRT history	(*n* = 15)				
	8 (53.3%)	4 (17.4%)	7 (38.9%)	3 (25.0%)	
Years on systemic HRT	0.50 (*n* = 15)	0.00	0.00	0.00	0.055
No OCP History	(*n* = 14)	(*n* = 26)	(*n* = 18)		
	6 (42.9%)	9 (40.9%)	7 (43.8%)	6 (50.0%)	
Years on OCP	1.25 (*n* = 14)	2.00 (*n* = 21)	4.50 (*n* = 16)	0.50	0.839
Comorbidities					
Type 2 Diabetes	0 (0.0%)	5 (21.7%)	0 (0.0%)	4 (33.3%)	
Hyperlipidemia	5 (29.4%)	6 (26.1%)	2 (11.1%)	8 (66.7%)	
Hypertension	6 (35.3%)	18 (78.3%)	1 (5.6%)	6 (50.0%)	
Hypothyroidism	4 (23.5%)	3 (13.0%)	4 (22.2%)	2 (16.7%)	
Regular Medications	12 (70.6%)	19 (82.6%)	11 (61.1%)	9 (75.0%)	
Tobacco Use		(*n* = 21)		(*n* = 10)	
Never	10 (58.8%)	11 (52.4%)	16 (88.9%)	8 (80.0%)	
Current	2 (11.8%)	2 (9.5%)	0 (0.0%)	1 (10.0%)	
Previous	5 (29.4%)	8 (38.1%)	2 (11.1%)	1 (10.0%)	
Pack Years	0.00	0.00 (*n* = 21)	0.00	0.00 (*n* = 10)	0.171
Glucose (mmol/L)	4.70 ^c^	5.20 (*n* = 23)	5.10	5.60 ^c^	0.013
Insulin (μU/mL)	4.30 ^d^ (*n* = 15)	9.50 ^de^ (*n* = 22)	4.80 ^e^ (*n* = 15)	7.70 (*n* = 10)	0.002
HbA1c IFCC (mmol/mol)	37.0 (*n* = 16)	39.0 ^f^	34.5 ^fg^	40.0 ^g^	0.003
HbA1c NGSP (%)	5.55 (*n* = 16)	5.80 ^h^	5.35 ^h^	5.80	0.005
HDL (mmol/L)	1.90 (*n* = 16)	1.30 (*n* = 20)	1.65	1.30	0.043
LDL (mmol/L)	2.80 (*n* = 16)	2.85 (*n* = 20)	2.45	1.90	0.233
Triglycerides (mmol/L)	1.45 (*n* = 8)	1.35 (*n* = 20)	1.30 (*n* = 15)	1.60 (*n* = 11)	0.554
CRP (mg/L)	1 ^i^ (*n* = 16)	4 ^ij^ (*n* = 22)	1.5 ^j^	3	0.003
Estradiol (pmol/L)	19.0 ^k^ (*n* = 13)	39.5 ^kl^ (*n* = 20)	16.0 ^l^ (*n* = 17)	31.0 ^g^ (*n* = 11)	0.005

^a^ Pairwise comparison revealed a significant difference (*p* = 0.004^#^). ^b^ Pairwise comparisons revealed a significant difference between lean control group and obese control group (*p* = 0.002^#^), lean control group and obese cancer group (*p* = 0.000^#^), lean cancer group and obese control group (*p* = 0.002^#^), and lean cancer group and obese cancer group (*p* = 0.000^#^). ^c–l^ Pairwise comparisons revealed a significant difference (*p* < 0.05^#^) and ^m^ *p* values were calculated using the Kruskal-Wallis H Test, and # *p* values have been adjusted by the Bonferroni correction for multiple comparison tests. HRT (hormone replacement therapy), OCP (oral contraceptive pill), HDL (high-density lipoprotein), LDL (low-density lipoprotein), HbA1c (hemoglobin A1c) International Federation of Clinical Chemistry (IFCC), HbA1c National Glycohemoglobin Standardization Program (NGSP), C-reactive protein (CRP).

**Table 2:** Mouse uterine pathology.

			Pathology Results, *n* (%)			
Genotype	Diet	Mice (*n*)	Normal	Hyperplasia	Atypical Hyperplasia	Cancer
*Lkb1^+^* ^/+^	Chow	4	1 (25%)	2 (50%)	1 (25%)	
*Lkb1^+^* ^/+^	Western	4		1 (25%)	3 (75%)	
*Sprr2f-Cre; Lkb1^L^* ^/+^	Chow	24	3 (12.5%)	6 (25%)	14 (58.3%)	1 (4.2%)
*Sprr2f-Cre; Lkb1^L^* ^/+^	Western	29	8 (27.6%)	7 (24.1%)	13 (44.8%)	1 (3.5%)

Statistical analyses display that for all genotypes combined, there is no significant difference in Chow vs. Western/Normal vs. Disease contingency (*p* = 0.52 Fisher’s Exact) or Chow vs. Western/Normal vs. Hyperplasia vs. Atypical Hyperplasia (no cancer) Chi-square = 0.95, *p* = 0.62. *Lkb1* (liver kinase B1), *Cre* (cre recombinase), *Sprr2f* (small proline-rich protein 2F), *Lkb1^+^*^/+^ (mice are wild-type for LKB1 in the endometrium), *Sprr2f-Cre; Lkb1^L^*^/+^ (mice are heterozygous for LKB1 in the endometrium).

## Data Availability

The 16S rRNA amplicon sequencing data generated within this manuscript were submitted to the European Nucleotide Archive under the accession number PRJEB50483.

## Supplementary Materials

The following supporting information can be downloaded at: https://www.mdpi.com/article/10.3390/cancers14092141/s1, Figure S1: Microbiota detection in samples versus the negative controls; Figure S2: Alpha diversity measures of endometrial microbiota in humans; Figure S3: Validation of the *Sprr2f-Cre: LKB1^L/L^* mouse model of endometrial cancer backcrossed to C57BL/6J mice. Figure S4: Endometrial microbiota of mice on chow or a western diet.
