# Supplementary material for: Differences in the Active Endometrial Microbiota across Body Weight and Cancer in Humans and Mice
Source: Cancers (Basel). 2022 Apr 25;14(9):2141. doi: 10.3390/cancers14092141 (PMC9100094; doi:10.3390/cancers14092141)
Supplement: Supplementary file 1 [file cancers-14-02141-s001.zip › cancers-1679327-supplementary.pptx]

## Slide 1
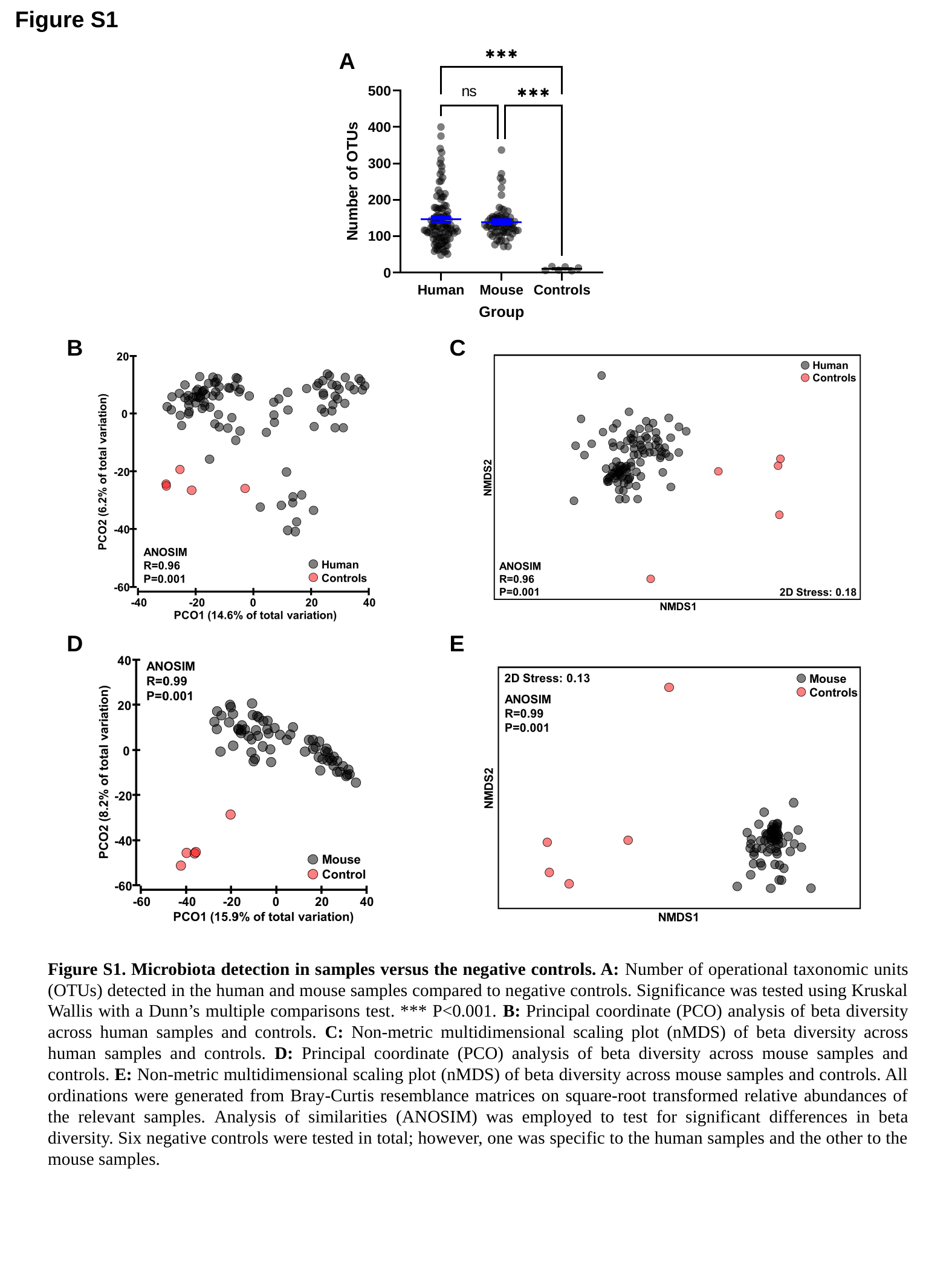

Figure S1
A
B
C
D
E
Figure S1. Microbiota detection in samples versus the negative controls. A: Number of operational taxonomic units (OTUs) detected in the human and mouse samples compared to negative controls. Significance was tested using Kruskal Wallis with a Dunn’s multiple comparisons test. *** P<0.001. B: Principal coordinate (PCO) analysis of beta diversity across human samples and controls. C: Non-metric multidimensional scaling plot (nMDS) of beta diversity across human samples and controls. D: Principal coordinate (PCO) analysis of beta diversity across mouse samples and controls. E: Non-metric multidimensional scaling plot (nMDS) of beta diversity across mouse samples and controls. All ordinations were generated from Bray-Curtis resemblance matrices on square-root transformed relative abundances of the relevant samples. Analysis of similarities (ANOSIM) was employed to test for significant differences in beta diversity. Six negative controls were tested in total; however, one was specific to the human samples and the other to the mouse samples.

## Slide 2
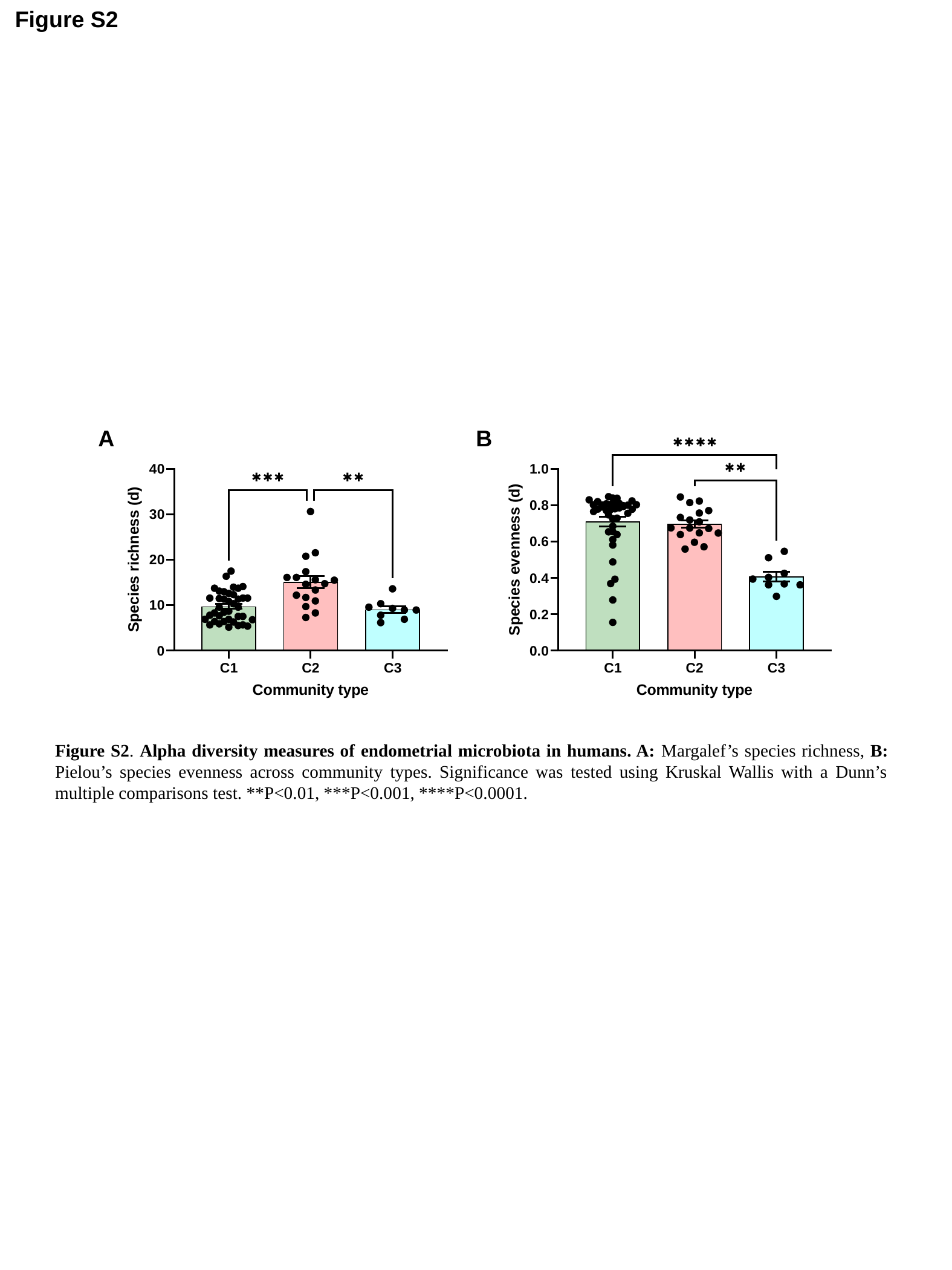

Figure S2
A
B
Figure S2. Alpha diversity measures of endometrial microbiota in humans. A: Margalef’s species richness, B: Pielou’s species evenness across community types. Significance was tested using Kruskal Wallis with a Dunn’s multiple comparisons test. **P<0.01, ***P<0.001, ****P<0.0001.

## Slide 3
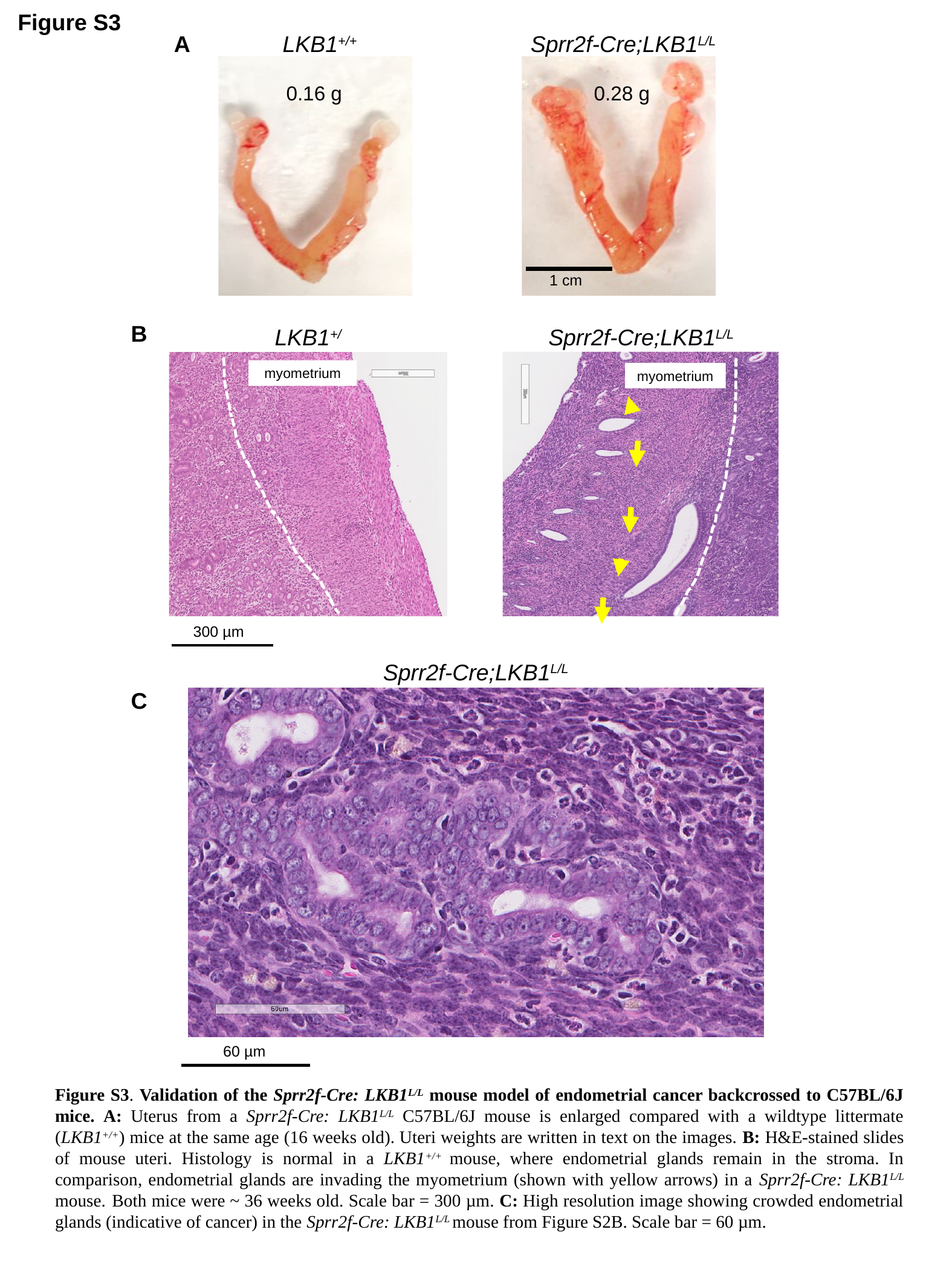

Figure S3
A
Sprr2f-Cre;LKB1L/L
LKB1+/+
0.28 g
0.16 g
1 cm
B
LKB1+/+
Sprr2f-Cre;LKB1L/L
myometrium
myometrium
300 µm
Sprr2f-Cre;LKB1L/L
C
60 µm
Figure S3. Validation of the Sprr2f-Cre: LKB1L/L mouse model of endometrial cancer backcrossed to C57BL/6J mice. A: Uterus from a Sprr2f-Cre: LKB1L/L C57BL/6J mouse is enlarged compared with a wildtype littermate (LKB1+/+) mice at the same age (16 weeks old). Uteri weights are written in text on the images. B: H&E-stained slides of mouse uteri. Histology is normal in a LKB1+/+ mouse, where endometrial glands remain in the stroma. In comparison, endometrial glands are invading the myometrium (shown with yellow arrows) in a Sprr2f-Cre: LKB1L/L mouse. Both mice were ~ 36 weeks old. Scale bar = 300 µm. C: High resolution image showing crowded endometrial glands (indicative of cancer) in the Sprr2f-Cre: LKB1L/L mouse from Figure S2B. Scale bar = 60 µm.

## Slide 4
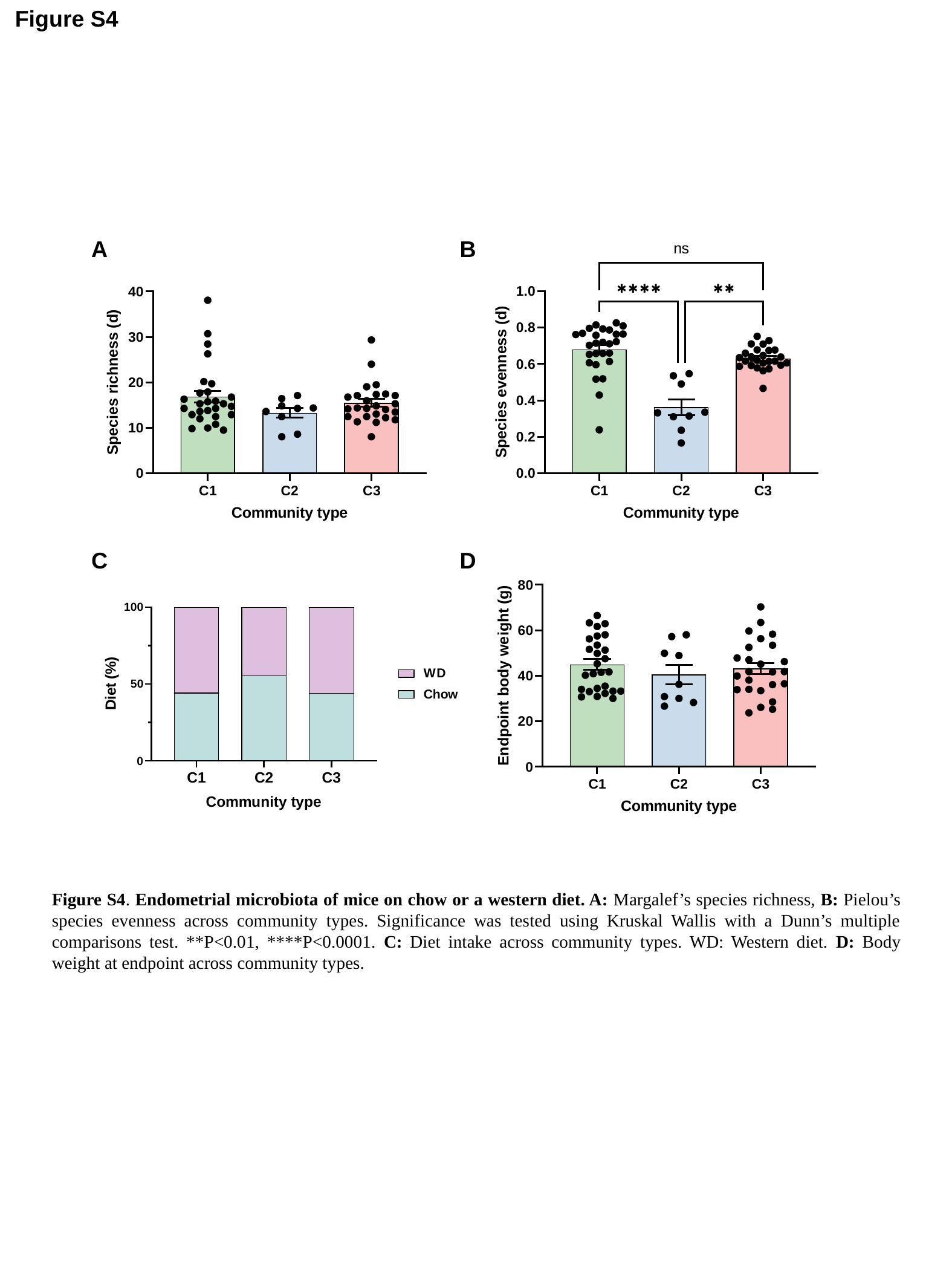

Figure S4
A
B
C
D
Figure S4. Endometrial microbiota of mice on chow or a western diet. A: Margalef’s species richness, B: Pielou’s species evenness across community types. Significance was tested using Kruskal Wallis with a Dunn’s multiple comparisons test. **P<0.01, ****P<0.0001. C: Diet intake across community types. WD: Western diet. D: Body weight at endpoint across community types.
